# Contrast and Strength of Visual Memory and Imagery Differentially Affect Visual Perception

**DOI:** 10.1371/journal.pone.0084827

**Published:** 2013-12-20

**Authors:** Elyana Saad, Juha Silvanto

**Affiliations:** 1 Brain Research Unit, O.V. Lounasmaa Laboratory, School of Science, Aalto University, Espoo, Finland; 2 Institute of Behavioral Sciences, University of Helsinki, Helsinki, Finland; 3 Department of Psychology, Faculty of Science and Technology, University of Westminster, London, United Kingdom; Radboud University Nijmegen, Netherlands

## Abstract

Visual short-term memory (VSTM) and visual imagery have been shown to modulate visual perception. However, how the subjective experience of VSTM/imagery and its contrast modulate this process has not been investigated. We addressed this issue by asking participants to detect brief masked targets while they were engaged either in VSTM or visual imagery. Subjective experience of memory/imagery (strength scale), and the visual contrast of the memory/mental image (contrast scale) were assessed on a trial-by-trial basis. For both VSTM and imagery, contrast of the memory/mental image was positively associated with reporting target presence. Consequently, at the sensory level, both VSTM and imagery facilitated visual perception. However, subjective strength of VSTM was positively associated with visual detection whereas the opposite pattern was found for imagery. Thus the relationship between subjective strength of memory/imagery and visual detection are qualitatively different for VSTM and visual imagery, although their impact at the sensory level appears similar. Our results furthermore demonstrate that imagery and VSTM are partly dissociable processes.

## Introduction

Visual short-term memory (VSTM) and visual imagery are believed to involve the same mechanisms in the visual system that encode incoming visual information (e.g. 1-6). One consequence of this overlap is that VSTM and mental imagery can modulate the detection of concurrently viewed visual stimuli. Visual imagery has been shown to interfere with performance in various concurrent visual tasks, a phenomenon known as the Perky effect [7]. In these studies, participants are asked to perform a visual task involving detection or discrimination either with or without imagery; performance is generally worse in the imagery condition relative to the baseline condition [7-9]. The Perky effect, which occurs when the mental image and the visual target spatially overlap, has been explained in terms of imagery reducing target energy in the region of the visual field in which the image is located [8]. In this view, the interference occurs at early levels of visual processing. The disruptive effect of imagery on vision has also been proposed to reflect competition for a limited pool of resources to be shared between visual processing and the maintenance of the mental image (e.g. 10). Thus both perceptual and attentional mechanisms may account for these effects. Although facilitations of performance by imagery are sometimes found, these have been explained in terms of priming and bias effects [11].. In contrast, VSTM has been shown to *facilitate* participants’ sensitivity to detect incoming visual information, particularly when VSTM content matches the visual target [12,13]. For example, engagement in VSTM decreases reaction times to features of visual targets that are congruent with VSTM content [13]. This facilitation has been explained in terms of VSTM boosting visual processing by enhancing the baseline activation level of early perceptual representations (see e.g. 12,13). 

While previous studies have investigated the overall impact of memory and imagery on visual encoding, they have not considered how trial-by-trial variations in subjective experience of VSTM/imagery modulate these effects. On one hand, these effects may be independent of subjective experience, if they reflect phenomena occurring at the earliest levels of visual processing [14]. In this case, one would predict no relationship between the subjective measures of VSTM/imagery and visual detection. On the other hand, strong subjective experience might enhance the effects of VSTM and imagery on visual detection, if it reflects the strength of the underlying VSTM/imagery representation. In this case, one would expect subjective strength of VSTM to positively correlate with visual detection (as VSTM has been shown to facilitate visual detection, cf. [13]), whereas a subjectively strong mental image would be associated with reduced ability to detect the visual target (as imagery generally impairs visual perception (e.g. 8-10).

Here we investigated this issue by asking participants to detect a brief, masked sinusoidal, luminance-modulated grating (present on 50% of trials) while being engaged either in VSTM maintenance or visual imagery of a similar grating. Subjective experience of memory/imagery was assessed on a trial-by-trial basis by asking participants to report the strength of their memory/mental image on a scale from 1 to 9. In addition, we assessed the visual quality of the memory/imagery content by asking participants to match the contrast of their memory/mental image to exemplars of gratings presented at the end of the trial. Specifically, participants were asked to select the test grating which was the closest match to the contrast of their memory/mental image. We focused on luminance contrast as it has been shown to affect excitability of the visual cortex, such that mental images of high contrast increase visual cortical excitability more than those of low contrast [14]. In additional experiments we assessed the impact of VSTM (Experiment 2) and imagery (Experiment 3) on visual detection when the VSTM/imagery contents and the visual target were of different stimulus class. In these experiments, VSTM/imagery involved a colored shape while the visual target was the same (grating) as in Experiment 1. 

## Methods

### Participants

42 participants (18 female; mean age 24 years old) with normal or corrected to normal vision participated in the three experiments. All participants were naïve to the aim of the study, provided written informed consent before the experiment, and were monetary rewarded. The study was performed in agreement with the Declaration of Helsinki and approved by the ethics committee of the Hospital District of Helsinki and Uusimaa.

### Stimuli

Stimuli and task were controlled by E-prime v2.0 (Psychology Software Tools Inc., Pittsburgh, USA; http:// www.pstnet.com/eprime.cfm). All stimuli were sinusoidal luminance- modulated gratings (with a diameter of 5 degrees of visual angle; generated with Matlab), presented foveally from a viewing distance of 57 cm on a gray background. The spatial frequency of the gratings was 1.44 cycles/degree. Memory/imagery cues had a Michelson contrast of 0.3 whereas visual targets contrast’s was 0.12. The mask was a uniformly black circle with the same diameter as the gratings. The stimuli were presented on a 15-inch screen with 1024 × 768 pixel resolution. 

### Experimental sessions

#### Experiment *1*: the impact of VSTM and imagery on visual detection of targets from the same stimulus class

Two conditions were carried out for each participant, in separate sessions, and randomized session order. These involved the following behavioral manipulations:

1) VSTM condition

participants were instructed to hold the cue in memory during the maintenance period while looking at a fixation point in the middle of the screen.

2) Imagery condition

participants were asked to form a mental image of the cue and project it onto the fixation point in the middle of the screen during the maintenance period.

#### Experiment *2*: the impact of VSTM maintenance of shape information on visual detection of gratings

The aim of Experiment 2 was to assess the impact of VSTM and its subjective strength on detection when the VSTM content and the visual target are of different stimulus class. In this experiment, VSTM involved the maintenance of shape information (size and color of a rectangle), while the visual target was the same as in Experiment 1.

#### Experiment *3*: the impact of mental imagery maintenance of shape information on visual detection of gratings

The aim of Experiment 3 was to assess the impact of imagery and its subjective strength on detection when imagery content and the visual target are of different stimulus class. Imagery involved a visual shape (as in Experiment 2), while the visual target was the same as in Experiment 1.

### Experiment 1: Procedure

**Figure 1:**
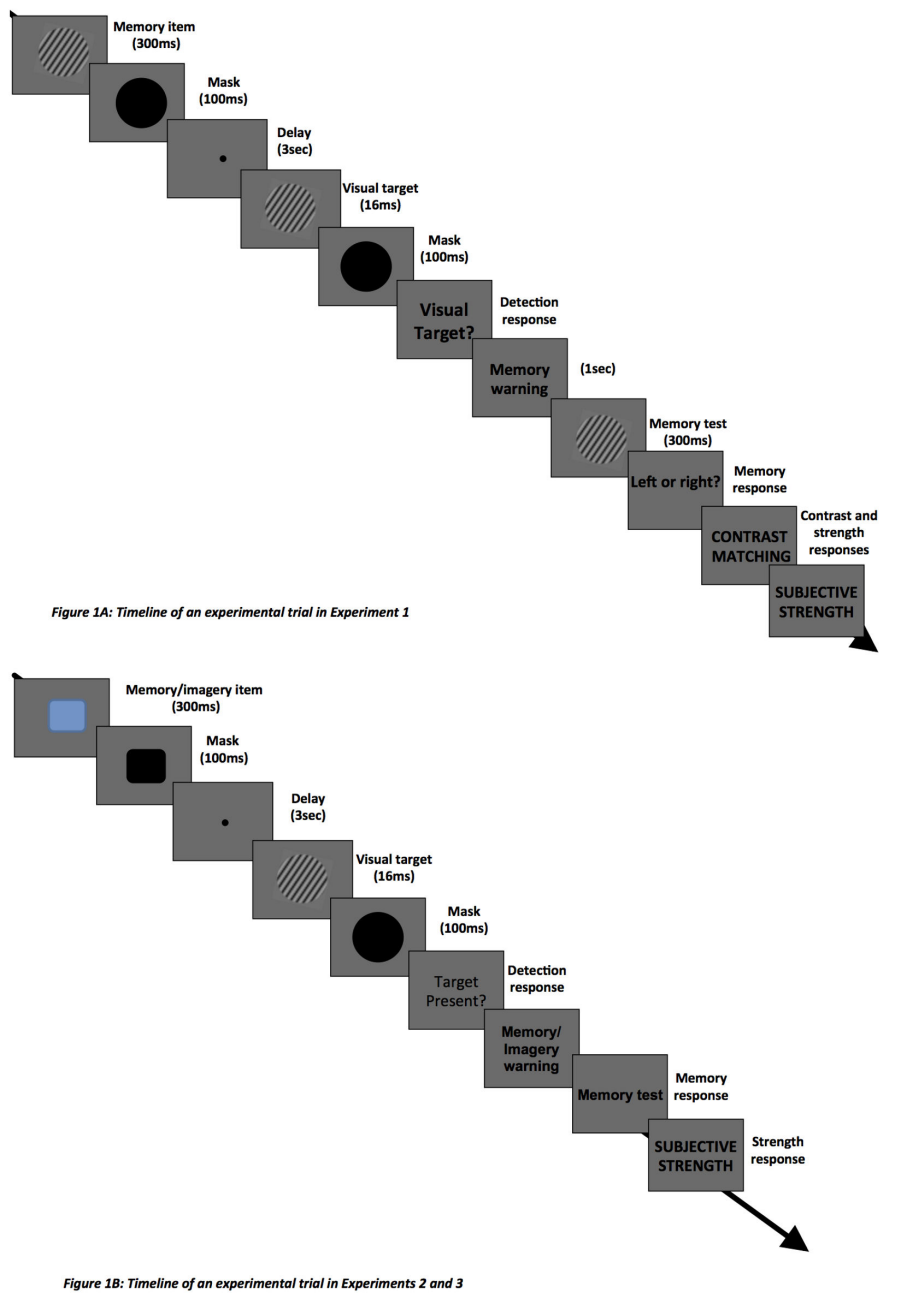
Timeline of an experimental trial. At the start of each trial, participants were shown a grating, which they needed either to hold in memory (VSTM condition) or to project as a mental image on the computer screen at fixation (imagery condition). During the maintenance, a masked grating was presented on 50% of trials; participants were asked to indicate whether or not they had perceived it. They were then asked to perform an orientation discrimination judgment based on the memory/imagery item; this involved indicating whether a test stimulus was tilted to the left or right relative to the memory/imagery cue. At the end of each trial, participants were asked to provide a rating of the strength of their memory or imagery on a 1-9 scale. In addition, they were asked to match the contrast of the grating held by memory/imagery to the exemplars presented on the screen. In Experiments 2 and 3, VSTM/imagery involved colored shapes (rectangle) while the visual target was the same as in Experiment 1 (see figure 1B).

Figure 1A shows the timeline of an experimental trial. Each trial began with a 1 sec foveal fixation point, followed by a memory cue (a grating tilted either +/-20, 30 or 40 degree from the vertical). To avoid afterimage induction by this cue, a mask (a uniformly black circle, appeared after the offset of the memory/imagery cue for 100 ms). In the VSTM condition, participants were instructed to hold the cue in memory; in the imagery condition, to form a mental image of the cue (see above). After a 3-second maintenance period, a visual target probe appeared on 50% of trials for 16 ms (a grating tilted either +/- 20, 30 or 40 degree from the vertical), followed by a mask (black circle presented for 100 ms). Participants were asked to report whether or not they perceived the visual target. The orientation of the visual target was either the same as that of the memory/cue, or of different sign (e.g. -20 deg memory cue followed by +20 deg target). This was followed by a memory test (a grating tilted either 10 degrees to the left or right from the memory/imagery). Participants were requested to judge the direction of the tilt with a button press (1=leftwards; 2=rightwards).

At the end of each trial, in order to assess *subjective experience*, participants were asked to provide a rating of the strength of their memory or imagery on a 1-9 scale (where 1 refers to the absence of any memory/mental image and 9 represents a memory/mental image that is as strong and clear as the memory cue). In the VSTM condition, participants were requested to rate their memory for the memory cue; in the imagery condition, they were asked to rate their mental image. We also assessed *the visual quality of the memory/imagery conten*t by asking participants to match the contrast of their memory/mental image to exemplars presented at the end of the trial. Specifically, participants were shown a display with 6 gratings and asked to choose the closest match to the contrast of their memory/mental image. The Michelson contrasts of these stimuli were: 0.05, 0.10, 0.15, 0.20, 0.25, and, 0.30. (Note that the contrast of the memory/imagery cue was always 0.30).

To measure the detectability of the visual target without any VSTM/imagery demand, in each session we included trials in which the memory cue was replaced by a black circle (50% of trials). These “baseline” trials were randomly mixed with the VSTM/imagery trials. Each participant completed 2 sessions (VSTM, imagery). Each session was run in 3 blocks, with each block consisting of 96 trials.

### Experiments 2 and 3: Procedure

To investigate how VSTM/imagery affects detection when the two involve items of different stimulus classes, we carried out two different experiments (Experiments 2 & 3) in which participants were required to maintain/imagine the shape and color of a rectangle (see Figure 1B). The memory/imagery cue was a square which was either blue or red and either small (diameter 4.5 deg of visual angle) or large (6.5 deg of visual angle) on each trial. In the memory test participants were asked to indicate whether the memory/imagery cue was: 1) blue and small; 2) blue and large; 3) red and small; 4) red and large. (While this task can be accomplished with verbal cues, it nevertheless fulfills the purpose of assessing the specificity of effects reported in Experiment 1.) In half of the trials a black circle replaced the rectangles. These trials assessed target detection without any VSTM/imagery demand. At the end of each trial, participants were asked to report the strength of their memory/mental image on a 1-9 scale, as in Experiment 1 (where 1 refers to the absence of any memory/mental image and 9 represents a memory/mental image that is as strong and clear as the memory/imagery cue). The contrast matching scale was not used in these experiments, as the contrast of the memory/imagery item was not manipulated. Experiment 2 and 3 were run separately. Each session was run in 3 blocks, with each block consisting of 96 trials.

## Results

### Experiment 1

#### Overall effects of VSTM and imagery on sensitivity and criterion

**Figure 2:**
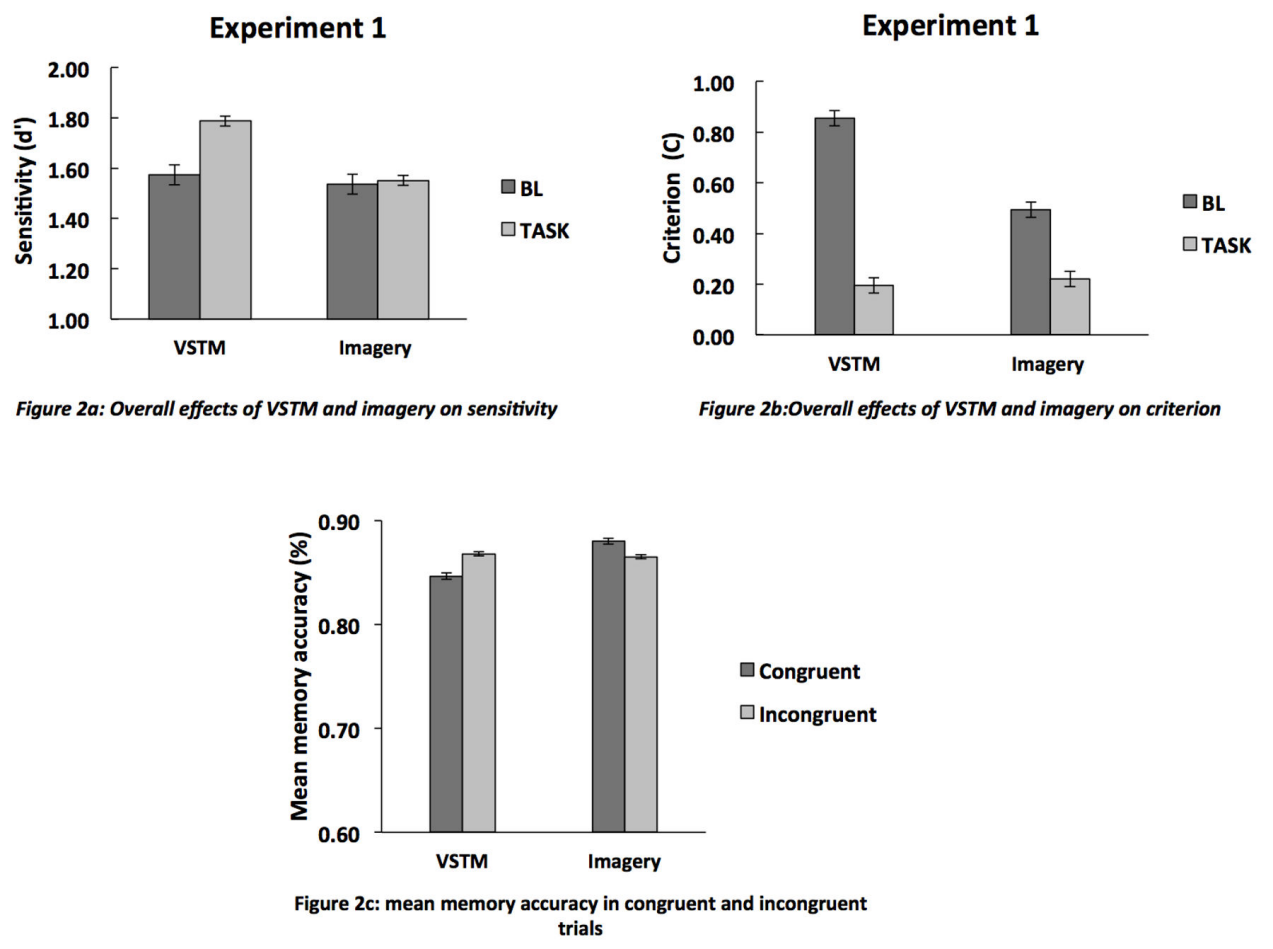
The mean (n=14) sensitivity (d’) and bias in each condition. In the “BL” condition, the memory cue was not shown and no VSTM or imagery was required. In the “task” condition, participants were engaged either in VSTM or in visual imagery. A) Engagement in VSTM increased visual detection sensitivity relative to baseline trials but not significantly; no such effect was found for imagery. B) Engagement in VSTM decreased the bias; a similar but nonsignificant trend was present in the imagery condition. C) The mean memory accuracy in congruent and incongruent trials. Detection performance is plotted by the function of VSTM/imagery cue-target orientation congruency. The Error bars indicate SDs from which between-subjects variance has been removed [15].

We first carried out a signal-detection analysis to calculate the overall sensitivity (d’) and criterion for each experimental condition; these are shown in Figure 2. Three participants were removed due to a mean detection sensitivity at baseline greater than two standard deviations above the group mean. These participants were replaced by the recruitment of three further participants. A repeated measure 2x2 ANOVA for sensitivity with task (VSTM, imagery) and condition (BL, task) revealed no significant effects of either task (F(1,13)=1.01; p=0.33; partial η2=0.07; observed power =0.15), nor condition (F(1,13)=1.61; p= 0.23; partial η2=0.11; observed power = 0.22) and no interaction (F(1,13)=1.10; p= 0.31; partial η2=0.08; observed power = 0.16). Thus the ANOVA revealed no significant effect of VSTM/imagery on detection, although an uncorrected pairwise comparison suggests a weak facilitation for VSTM (t(13) =-2.30; p=0.04).

A repeated measure 2x2 ANOVA for bias with task (VSTM, imagery) and condition (BL, task) revealed a significant effect of condition (F(1,13)=14.13; p= 0.002; partial η2=0.52; observed power=0.93). The main effect of task was not significant (F(1,13)=1.60; p=0.23; partial η2=0.11; observed power=0.22), and neither was the interaction (F(1,13)=2.33; p= 0.15; partial η2=0.15; observed power = 0.29.). Pairwise comparisons (t-tests) revealed a significant decrease in criterion (see Figure 2B) was observed in the VSTM condition (t(13) =3.50; p=0.004). This decrease was not statistically significant for imagery (t(13) =1.65; p=0.12).

The orientation of the visual target was either identical to that of the memory/imagery cue, or of opposite sign (i.e. memory cue tilted 20 degrees *leftwards* from the vertical was followed by a visual target tilted 20 deg *rightwards* from the vertical). We assessed whether this congruency affected detection. Figure 2C shows the proportion of hits as a function of stimulus congruency. There was no difference in the amount of hits between the congruent and incongruent conditions for neither VSTM (t(13)=-0.90; p=0.40) nor for imagery (t(13)=0.71; p=0.50). The mean memory performance for VSTM was 85% (SD = 0.07) on congruent trials vs 87% (SD = 0.07) on incongruent ones. The mean performance on memory trials in the imagery conditions was 88% (SD = 0.07) on congruent trials and 87% (SD = 0.06) on incongruent trials.

#### Relationship between memory/mental image contrast and detection performances

**Figure 3:**
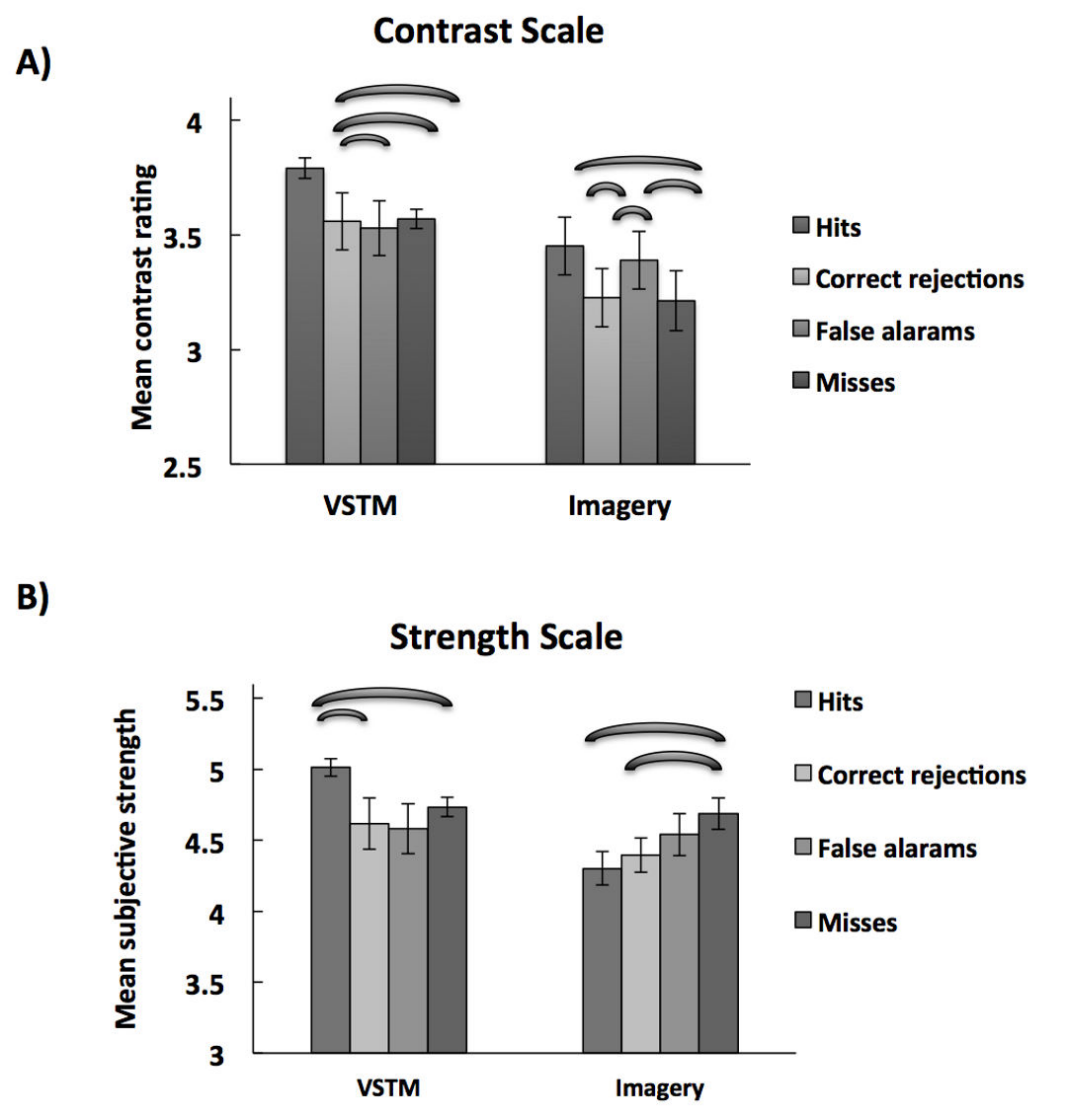
Relationship between VSTM/imagery contrast/subjective strength and visual detection. The Error bars indicate SDs from which between-subjects variance has been removed [15]. A) Mean (n=14) *visual* *contrast* *of* *the* *memory/mental* *image* on the contrast response scale as a function of performance in the detection task For VSTM, contrast was significantly higher for “hits” than for other trial types. For imagery, contrast was significantly higher for “hits” and “false alarms” than for “misses” and “correct rejections”. B) Mean (n=14) *subjective* *strength* *of* *the* *memory/mental* *image* as a function of performance in the detection task. For VSTM, subjective strength was significantly higher for “hits” than for other trial types. For imagery, subjective strength was significantly higher for “misses” and “false alarms” than for “hits” and “correct rejections”.

Figure 3A shows the mean visual contrast of the memory/mental image on the contrast response scale as a function of performance in the detection task. Trials on the detection task have been subdivided into *hits, misses, correct rejections* and *false alarms*. (Note that only trials on which participants performed correctly in the memory test for orientation were included in the analysis). A 2x4 ANOVA with task (VSTM, imagery) and trial type (Hits, misses, correct rejections, and false alarms) revealed a significant main effect of task (F(1,13) =43.66; p= 0.03; partial η2=0.31; observed power=0.61), and of trial type (F(7,12)=9.74; p=0.02; partial η2=0.22; observed power=0.77) and a close to significant interaction between the two (F(3,39) =9.83; p=0.06; partial η2=0.17; observed power=0.60). For VSTM, planned pairwise comparison revealed that the contrast of the memory item was higher on “Hits” than on other trial types (*vs. misses*: (t(13)=2.15; p=0.03); *vs. correction rejections*: (t(13) =2.20; p=0.04); *vs. false alarms*: (t(13)=2.88; p=0.01)). For imagery, Hits and false alarms were associated with higher mental image contrasts than misses and correct rejections (pairwise comparisons: *hits vs misses*: (t(13) =2.60; p=0.02); *hits vs correct rejections*: (t(13) =2.91; p=0.01); *false alarms vs. misses*: (t(13) =-2.24; p=0.04); *false alarms vs. correct rejections*: (t(13) =-2.15; p=0.05)). In other words, trials on which participants tended to report target presence (regardless of whether or not it was there) were associate with higher mental image contrast.

This result can be summarized as follows: for both VSTM and imagery, memory/imagery contrast was positively associated with the ability to correctly report target presence (hits), such that the reported contrast was higher for hits than for misses. For imagery, this applied also for false alarms, indicating that participants were biased to report target presence when the mental image contrast was high.

#### Relationship between subjective memory/imagery strength and detection performances

Figure 3B shows the mean subjective strength of the memory/mental image as a function of performance in the detection task. A repeated measure 2x4 ANOVA with task (VSTM, imagery) and trial type (Hits, misses, correct rejections, and false alarms) was carried out. This revealed a significance interaction between task and trial type (F (3,39)=3.41; p=0.03; partial η2=0.20; observed power=0.71). The effect of task (F(1,13) =6.24; p= 0.24; partial η2=0.10; observed power=0.20), and trial type (F(7,12) =6.24; p=0.10; partial η2=0.14; observed power=0.50) were not significant.

For VSTM, planned pairwise comparison revealed that the strength of the memory item was higher on “Hits” than on other trial types (*vs. misses*: (t(13)=2.16; p=0.05); *vs. correction rejections*: (t(13) =2.83; p=0.01); *vs. false alarms*: (t(13) =2.1; p=0.056). For imagery, the opposite pattern was observed: pairwise comparison revealed that the contrast of the mental image was higher on “Misses” than on “Hits” (t(13)=-3.21; p=0.007) and correction rejections (t(13) =-2.50; p=0.03). Also, strength of mental image was almost significantly higher on “False alarm” trials than on “Hits” (t(13) =-1.98; p=0.07). The difference between misses and false alarms was not significant (t(13) =1.39; p=0.19) (As above, only trials on which participants performed correctly in the memory test for orientation were included in the analysis).

This result can be summarized as follows: for VSTM, subjective strength is positively associated with the ability to correctly report target presence (hits). In contrast, for mental imagery, the pattern is the opposite: false responses (i.e. misses) are associated with higher subjective mental image strength than correct responses. Thus whereas memory strength aids detection, imagery strength interferes with it. 

### Experiment 2

#### Overall effects of shape VSTM on sensitivity and criterion

**Figure 4:**
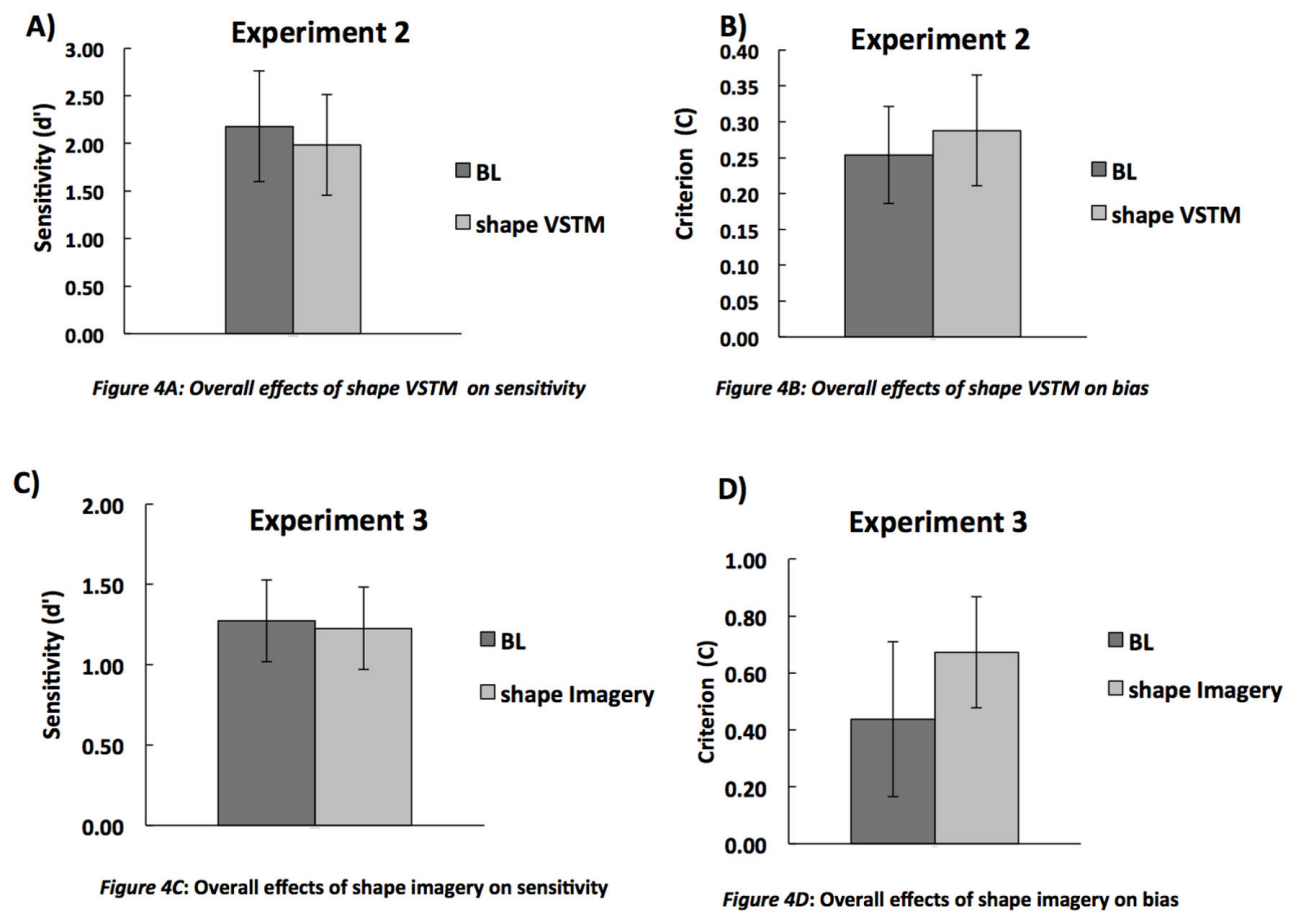
The mean (n=14) sensitivity (d’) and bias in Experiments 2 (panels A and B) and 3 (panels C and D). The Error bars indicate ± 1 SEM.

We carried a signal-detection analysis as in Experiment 1 to calculate the overall sensitivity (d’) and criterion; this is shown in Figure 4. Neither sensitivity (t(13) =2.30; p=0.08) nor criterion (t(13)=0.53; p=0.60) were affected by VSTM. The mean performance on shape memory trials was 94% (SD = 0.01). 

#### Relationship between subjective strength of VSTM and detection performance

**Figure 5:**
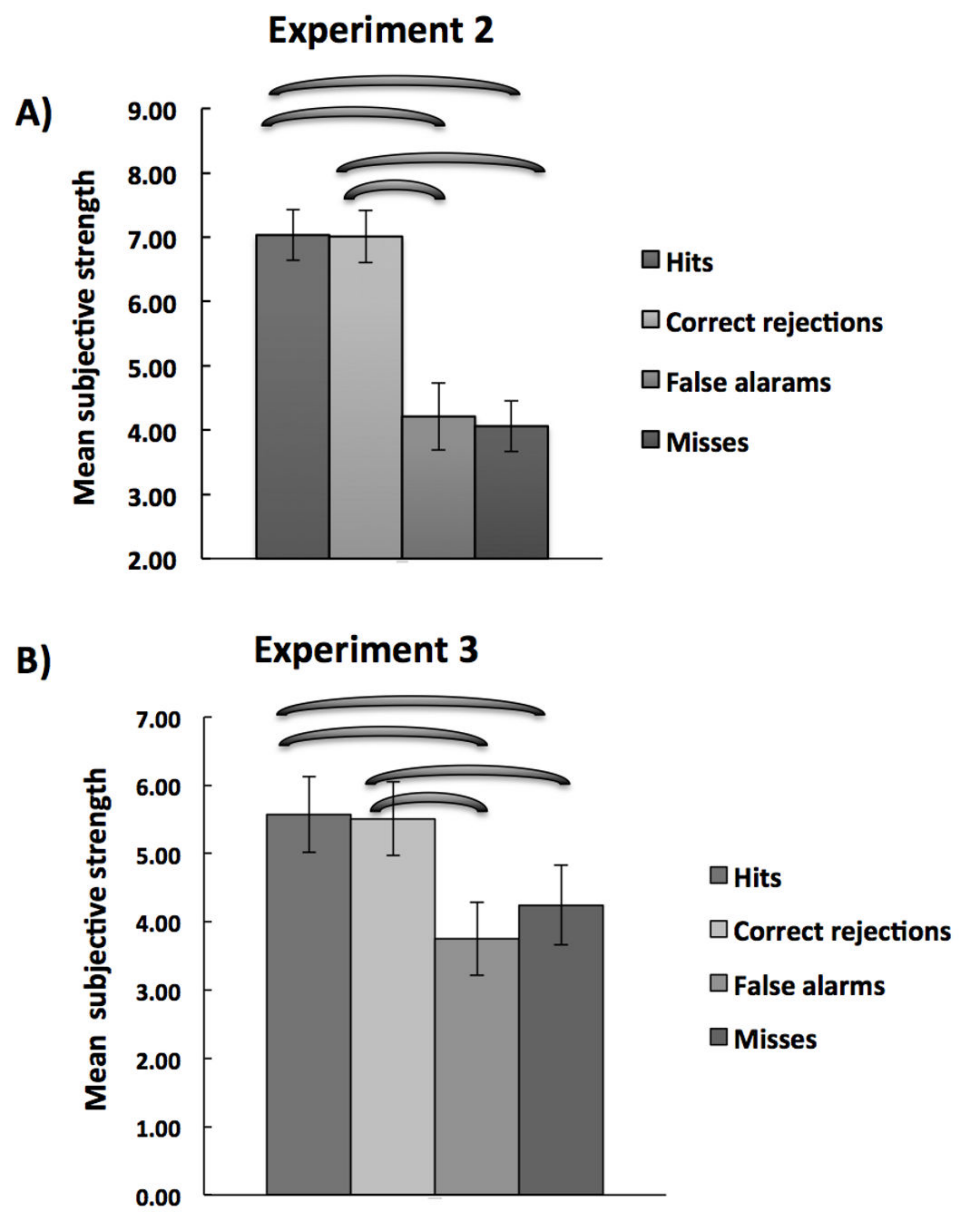
Mean subjective strength of VSTM in Experiment 2 (panel A) and imagery in Experiment 3 (Panel B) as a function of performance in the detection task. The Error bars indicate Error bars indicate ± 1 SEM.

Figure 5A shows the mean subjective strength of VSTM as a function of performance in the detection task. A repeated measure ANOVA with trial type (Hits, misses, correct rejections, and false alarms) as the main factor revealed a significance effect (F (1,13) =34.14; p<0.0001; partial η2=0.72; observed power = 1). 

Planned t-tests revealed that “Hits” and correct rejections were associated with higher subjective VSTM strength than misses and false alarms (*Hits vs. misses*: (t(13)=5.36; p=0.003); *Hits vs. false alarms*: (t(13) =4.59; p=0.002); Correct rejections vs. false alarms: (t(13)=4.54; p=0.001), correct rejections and misses: (t(13)=5.36; p=0.002). As in Experiment 1, only trials on which participants performed correctly in the memory test for shape were included in the analysis. In summary, trials on which participants tended to correctly report target presence and absence were associated with higher VSTM strength. 

### Experiment 3

#### Overall effects of shape imagery on sensitivity and criterion

We carried a signal-detection analysis as in Experiment 1 to calculate the overall sensitivity (d’) and criterion; this is shown in Figure 4C and 4D. Neither sensitivity (t(13) =0.24; p=0.81) nor criterion (t(13)=1.57; p=0.14) were affected by imagery. The mean performance on shape memory trials was 94% (SD = 0.01). The mean performance on imagery catch trials was 93% (SD = 0.04).

#### Relationship between subjective strength of imagery and detection performance

Figure 5B shows the mean subjective strength of imagery as a function of performance in the detection task. A repeated measure ANOVA with trial type (Hits, misses, correct rejections, and false alarms) as the main factor revealed a significance effect (F (3,39) =6.92; p=0.001; partial η2=0.35; observed power=0.97). Planned t-tests were carried out to examine this effect. Hits and correct rejections were associated with higher mental image strength than misses and false alarms (pairwise comparisons: *hits vs. misses*: (t(13) =2.38; p=0.05); *hits vs. false alarms*: (t(13) =3.71; p=0.005), *correct rejections vs. false alarms*: (t(13) =3.84; p=0.001), *correct rejections vs misses*: (t(13) =2.34; p=0.06). 

In summary, trials on which participants tended to correctly report target presence and absence were associated with higher mental image strength. In addition, holding in VSTM/imagery a stimulus of a different class than that of the visual target has different effects than those observed while maintaining and perceiving identical stimulus classes.

## Discussion

The central finding of the present study is that the subjective strength of VSTM and imagery modulate visual detection differently; subjective strength of VSTM was positively correlated with successful performance in a visual detection task, whereas the opposite pattern was found for imagery. For VSTM, trials on which the visual target was correctly detected (“hits”) were associated with higher subjective memory strength (strength scale), than trials on which participants failed to detect the target (“misses”). The same pattern was observed for the visual contrast of memory content (contrast scale): “hits” were associated with higher contrast than “misses”. Thus during VSTM maintenance, participants were more likely to detect the visual target when the subjective experience of memory strength as well as the contrast of the memory content were high. Overall, these results indicate that strong engagement in VSTM enhances the encoding of concurrently presented visual input (e.g. 12). It is important to note that only trials on which participants performed correctly in the memory test (which assessed the orientation of the memory cue) were included in the analyses; thus this effect cannot be explained merely in terms of VSTM fidelity modulating visual detection. In the general analysis, when detection performance was not analysed as a function of VSTM/imagery strength or contrast, engagement in VSTM/ imagery was not found to modulate visual sensitivity, although a clear trend was present for VSTM in Experiment 1, consistent with previous reports [12]. 

A key finding of the present study is that the impact of imagery strength on visual detection was the opposite of what was observed for VSTM. Subjective strength of imagery (strength scale) was *negatively* associated with visual detection, such that wrong responses (i.e. misses and false alarms) were associated with stronger mental image strength than correct responses (i.e. hits and correct rejections). In other words, participants were more likely to perform correctly in the detection task when the subjective strength of imagery was weaker. This can be interpreted in terms of competition for a limited pool of resources to be shared between visual processing and the maintenance of the mental image (e.g. 10); good performance in one leads to worse performance in the other. Therefore, whereas strong subjective experience of VSTM aids visual detection, imagery hinders it. 

Interestingly, subjective strength of visual imagery and the contrast of the mental image affected visual detection in opposite ways. Whereas the former was *negatively* associated with visual detection (as discussed above), the latter had a *positive* relationship with visual perception: the tendency to report the presence of a visual target (i.e. hits and false alarms) was associated with mental images of higher contrast than trials on which participants did not report target presence (i.e. misses and correct rejections). Whereas the strength scale could be perceived as a general measure of memory/imagery performance, which in addition to memory/imagery content may reflect number of variables such as participants’ experience of encoding and maintenance as well as attentional allocation, the contrast scale focused on a specific visual feature (contrast) of the memory/mental image. We focused on contrast because its modulation has been shown to modulate the excitability of the visual cortex in a positive fashion [14]. Specifically, incoming visual signal is more likely to reach perceptual threshold during maintenance of high contrast mental image, as in this circumstance even a weak input may be sufficient to push the activation level beyond threshold. In the imagery condition, the finding that *both* hits and false alarms were associated with mental imagery of higher contrast than misses and correct rejections is consistent with this. 

How can we interpret the differential effects of contrast and subjective strength of mental imagery on visual perception? One explanation can be made in terms of the level in the visual system where these effects take place. Whereas contrast is a low-level visual feature that is encoded in the early visual areas, the competition between visual processing and visual imagery for the limited pool of resources is likely to be resolved in higher-level areas. Therefore, while the engagement of the early visual areas by imagery may enhance their excitability to visual stimuli, this benefit is offset by the need to allocate attentional resources to the mental image. This may explain why visual imagery generally impairs visual perception (e.g. 8) even though at the sensory level it enhances sensitivity/susceptibility to external input (e.g. 16). In general terms, the relationship between subjective strength and visual detection found here is consistent with the hypothesis that strong subjective experience enhances the effects of VSTM and imagery on visual detection, which have been shown to be facilitatory for VSTM (e.g. 13] and disruptive for imagery (e.g. [8-10]). It needs to be noted however that significant facilitatory/disruptive effect of VSTM and imagery on overall detection performance were not found in the present study. 

The results of the control experiments (Experiments 2 and 3) indicate that, when the content of VSTM/imagery and visual target are of different stimulus classes, their subjective strength/detectability go hand-in hand. Specifically, correct responses in the detection task were associated with higher subjective strength of VSTM and imagery than incorrect responses. A simple explanation for this pattern of result is that it reflects trial-by-trial variability in attentional level of the participants. In some trials, participants are more focused on the tasks than in other trials, and perform well in both imagery/VSTM maintenance and detection. The important point is that VSTM/imagery and detection do not compete in these conditions. As noted above, such competition does take place for imagery when the stimuli are of the same class (as found in Experiment 1). 

In summary, our results show the following: for both VSTM and imagery, the contrast of the memory/mental image was positively associated with reporting target presence; thus at the sensory level, both VSTM and imagery appear to facilitate visual perception or increase the likelihood of stimulus presence being reported. However, a dissociation was found in the relationship between the subjective experience of memory/imagery strength and visual detection: subjective strength of VSTM was positively associated with visual detection whereas the opposite pattern was found for imagery. Moreover, this dissociation was not found when the memory/imagery cues were of different stimulus class than the visual target. Thus the relationship between subjective experience of memory/imagery and visual detection are qualitatively different for VSTM and visual imagery, although their impact at the sensory level appears to be similar.
